# Summary Measures of Adherence Using Pill Counts in Two HIV Prevention Trials: The Need for Standardisation in Reporting

**DOI:** 10.1007/s10461-013-0542-9

**Published:** 2013-06-26

**Authors:** Kathy Baisley, Jared M. Baeten, James P. Hughes, Deborah J. Donnell, Jing Wang, Richard Hayes, Deborah Watson Jones, Connie Celum

**Affiliations:** 1 Department of Infectious Disease Epidemiology, London School of Hygiene and Tropical Medicine, London, UK; 2 Department of Global Health, University of Washington, Seattle, WA USA; 3 Department of Medicine, University of Washington, Seattle, WA USA; 4 Department of Epidemiology, University of Washington, Seattle, WA USA; 5 Department of Biostatistics, University of Washington, Seattle, WA USA; 6 Vaccine and Infectious Disease Division, Fred Hutchinson Cancer Research Center, Seattle, WA USA; 8 Statistical Center for HIV/AIDS Research & Prevention (SCHARP), Fred Hutchinson Cancer Research Center, Seattle, WA USA; 9 Department of Clinical Research, London School of Hygiene and Tropical Medicine, London, UK; 10 Mwanza Intervention Trials Unit, National Institute for Medical Research, Mwanza, Tanzania

**Keywords:** Adherence, HIV prevention, Pill counts

## Abstract

For trials of user-dependent HIV prevention products, accurate adherence measurements are essential to interpret and compare results across trials. We used pill count data from two recent HIV prevention trials of herpes simplex virus type 2 (HSV-2) suppression, to show that estimates of adherence vary substantially depending on assumptions that are made in analysing pill count data. We associate calculated adherence with biological markers of anti-HSV-2 activity. In both trials, calculated adherence varied considerably, depending on the summary measure used, and the handling of intervals with apparent ‘over-adherence’ (fewer pills returned than expected), and unreturned pills. Intervals of apparent over-adherence were associated with reduced antiviral effects on biological markers of herpes reactivation, indicating these are likely to represent periods of non-adherence. Our results demonstrate the clear need for standardisation in reporting of adherence data that are based on pill counts.

## Introduction

Novel, user-dependent, biomedical HIV prevention interventions require high adherence to achieve efficacy, as demonstrated in clinical trials of tenofovir gel [1, 2], daily oral antiretroviral (ARV) pre-exposure prophylaxis (PrEP) [2–6], and ARVs taken by HIV-positive persons to prevent transmission [7]. For such user-dependent methods, with dosing either at fixed intervals or linked to specific events (such as sex acts), accurate and objective measurements of adherence are critical for understanding trial results, since limited or zero effectiveness of an investigational product may be due to either the product’s lack of biological efficacy or sub-optimal user adherence [8–10]. Moreover, correlating the level of HIV protection to the level of adherence might provide valuable insight into the relationship between a product’s pharmacokinetic properties and its biologic activity [6, 11, 12]. Lastly, understanding patterns and correlates of adherence can inform the design of future prevention trials of similar interventions by providing realistic estimates of possible effect sizes based on achievable levels of adherence.

Various methods have been used to gather information on adherence in biomedical HIV prevention trials, including self-report by face-to-face [3, 6, 13] or computer-assisted [13] interview, participant diaries [14], drug dispensing records [3], electronic monitoring of dosing [4], drug levels in blood, urine or tissues [1, 3, 6] and biological markers, such as HIV plasma viral load in studies of ARVs to reduce HIV transmission [4]. The simplest and most commonly used measure for quantifying adherence to HIV prevention interventions has been to count unused study product (for example, remaining pills in trials of oral PrEP or unused applicators in microbicide trials) returned at scheduled study visits.

A challenge to interpreting and comparing trial results is the lack of standardization in defining, measuring, analysing and reporting adherence for HIV prevention trials. Trials often report simple summary measures of adherence, with heterogeneity in calculation of these summary measures [15–18]. The absence of a common reporting standard undermines interpretation of adherence patterns, product effectiveness, and predictors of adherence. Even with seemingly simple methods such as pill counts, adherence measurement is complex with issues concerning how to handle visits when pills are not returned, apparent ‘over-adherence’ (>100 % of expected pills for the interval between visits), missed visits (when product is thus not dispensed for a period) and protocol-specified time off treatment such as during pregnancy.

An additional challenge is the lack of a standardised adherence terminology with clear definitions to designate the same concepts across trials. Recent reviews have proposed a new taxonomy for adherence research, with uniform terms and definitions [19, 20]. ‘Adherence to medication’ describes the participant’s use of the study product as prescribed. Adherence has three components: initiation (the time point of first dose), execution (the extent to which the participant’s product use corresponds to the instructed regimen), and discontinuation (the time point when the participant stops using the product). A fourth term, persistence, describes the length of time between initiation and discontinuation.

We propose an additional term, adherence ‘coverage’, to describe a participant’s tablet taking during the entire time the participant is in a trial, including temporary treatment interruptions. This is to recognise that, although the participant may have been instructed to stop taking the drug (and is thus taking the product as prescribed), these periods should be included when reporting trial adherence. Adherence coverage is arguably the most relevant measure for interpreting trial results, as it provides an indication of whether sufficient study medication was taken to expect a biological response. We also propose the term ‘apparent over-adherence’ to describe periods when counts of returned study product suggest that the participant has taken >100 % of expected doses.

In this paper, we use data from two recent biomedical HIV prevention trials of herpes simplex virus type 2 (HSV-2) suppression, which both dispensed pills to be taken twice daily and used clinic-based pill counts of returned study product to measure adherence, to demonstrate how estimates of adherence vary depending on the assumptions that are made in analysing and reporting adherence data. In addition, we relate calculated adherence to biological markers of pill activity (genital herpes reactivation), to assess the value of pill counts as a measure of ‘true’ adherence.

## Methods

We used data from two double blind, placebo-controlled trials of daily HSV-2 suppressive therapy with acyclovir for HIV prevention: one in Mwanza, Tanzania [21] and HPTN 039, which was conducted in nine sites in Peru, South Africa, United States, Zambia, and Zimbabwe[22] (Table 1). Participants were randomised to twice daily acyclovir 400 mg or matching placebo. In both trials, there was no evidence of a difference in HIV incidence between the acyclovir and placebo arms overall, whilst among the sub-group with optimal adherence, those randomized to acyclovir had decreased HSV-2 activity (genital ulcers or HSV-2 DNA shedding) compared with those randomized to placebo [21, 22]. There was no evidence that adherence differed between randomization arms in either trial [22, 23]. In Mwanza, there was no evidence that participants knew their randomization assignment [24]. In HPTN 039, among the one-third of participants who thought that they knew their randomisation assignment in an assessment after the end of the study, a slightly higher proportion of participants in the acyclovir arm than in placebo perceived they were randomised to acyclovir [23].

**Table 1:** Summary of two herpes suppression trials for HIV prevention: Mwanza and HPTN 039

	Mwanza trial	HPTN 039
Study design		
Location	Tanzania	Peru, South Africa, USA, Zambia, Zimbabwe
Population	HIV negative and HIV positive women	HIV negative women and men who have sex with men
Number randomised	1,305	3,277 (1395 women, 1882 men)
Length of follow up	Up to 30 months	Up to 18 months
Frequency of scheduled visits	3 monthly	Monthly
Could resume tablets after treatment interruption	Yes, but not for pregnancy or if participant requested to stop taking the drug	Yes
Main endpoints	HIV incidence; genital and plasma HIV RNA in HIV/HSV-2 co-infected women	HIV incidence; incidence of HSV-2 ulcers
Adherence measurement for main trial publications		
Medication dispensed	Blister strips of 14 tablets each	Bottles of 70
Identification numbers on bottles/blister strips	Non-unique batch ID recorded when dispensed only	Yes, unique ID recorded when dispensed and returned
Amount of surplus tablets (‘buffer stock’) supplied at each visit	2–3 weeks	5 days
Pill counts		
Frequency	Every visit	Every visit
Performed by	Dispensing clinician	Dispensing clinician
Self report		
Frequency	Scheduled visits	Every visit
Questions used	If missed any tablets since last visit; if missed ≥3 consecutive days of tablets; total number of tablets missed	If missed any tablets since last visit; if missed ≥2 consecutive doses; maximum number of consecutive missed doses
Adherence calculations		
Handling of intervals with adherence >100 %	Allowed 1–4 tablets over^a^; if >4 tablets over, and did not report taking extra, classed as missing	Allowed 5 % over; if >5 % over, classed as missing
Handling of intervals when bottles/blister strips not returned	Classified as missing	Asked to return at next visit. If never returned, used self report. If self-report <100 %, classed as missing.
Returned pills matched to visit dispensed	No, assumed to have been dispensed at previous visit	Yes
Handling of intervals when adherence classed as missing	Replaced as 70 % of expected tablets taken, or all tablets dispensed, whichever was less	Dropped from numerator and denominator
Included periods off treatment	Yes, except for pregnancy	Yes
Summary measures used and reported adherence	Median adherence = 90 %; person–years with ≥90 % adherence = 52 % (acyclovir arm) and 51 % (placebo)	Median % of expected doses taken = 86 %; median % of dispensed drug taken = 94 %; % of quarterly visits with ≥90 % adherence = 73 %
Summary of data included in current analysis		
Total visits attended	9,199	48,446
Scheduled visits	9,139	47,551
Interim visits	60	895
Total visits analysed (i.e. excluding visits after pregnancy)	8,149	47,243
Participants with at least one pill count	1,242	3,140
Participants completing follow-up	972 (78 %)	2,428 (77 %)
Person–years of follow-up	2,144	4,081
Participants with treatment interruption	233 (19 %)^b^; 14 (1 %)^c^	183 (6 %)^b^; 48 (2 %)^c^
Person–years off treatment	0.73^c^	13^c^
Visits with ‘measurable’ adherence		
% visits with tablets returned on time	95 %^d^	89 %
% visits with late/unscheduled returns	3 %	7 %
Visits with ‘unmeasurable’ adherence (tablets never returned)		
% of all visits	2 %	4 %
% of participants ever	12 %	31 %
Apparent over-adherence (>105 %)		
% of all visits	19 %	11 %
Median (IQR) tablets over	28 (16–46)	6 (4–10)
% of participants ever	66 %	65 %

^a^In the calculations of adherence summary measures for this paper (Methods 1 and 2), we allowed up to 5 % over-adherence in Mwanza instead of 1-4 tablets over, for comparability with HPTN 039

^b^Participants with treatment interruptions for pregnancy (censored at pregnancy for adherence calculations)

^c^Participants with treatment interruptions not related to pregnancy

^d^In Mwanza, blister packs could not be matched to the visit at which they were dispensed because package numbers were not recorded at returns; assume any tablets returned were dispensed at previous visit

### Mwanza Trial

The Mwanza trial enrolled 1305 HSV-2 seropositive women aged 16–35 years working in bars, guesthouses and similar facilities in 19 communities in northwest Tanzania. The trial enrolled both HIV negative (821) and HIV positive (484) women, to examine the effect of acyclovir on HIV acquisition and among HIV-infected women, on HIV genital shedding and viral load. Women were followed every 3 months for 12–30 months. Women were withdrawn permanently from the study medication if they became pregnant, and study medication was withdrawn temporarily for medical reasons such as intercurrent illness. Women who were withdrawn from tablets continued to attend follow-up visits, unless they requested to withdraw completely from the trial.

At each quarterly visit, women were issued with a supply of tablets to last until their next scheduled visit, plus an additional 2–3 week buffer stock in case they were late. Tablets were supplied in blister strips of 14 tablets. The batch number was recorded when dispensed, but not when returned. At each visit, women were asked to bring unused tablets and empty blister strips from the preceding visit; the number of remaining tablets was recorded by the dispensing clinician. In addition to pill counts, women were asked about self-reported adherence.

### HPTN 039 Trial

The HPTN 039 trial enrolled 1,358 women and 1,814 men who have sex with men who were HSV-2 seropositive and HIV negative. Participants were followed monthly for 12–18 months with quarterly pregnancy and HIV testing. Women with positive pregnancy tests were withdrawn from study medication until pregnancy tests were negative, and then tablets were resumed. Women who were withdrawn from study medication continued to attend follow-up.

At each visit, participants were issued with a supply of tablets to last until their next visit, scheduled every 30 days, plus a 5-day buffer stock in case they were late. Tablets were packaged in bottles of 70; each bottle had a unique identification number that was recorded when dispensed and returned. At each visit, participants were asked to return the bottle and unused tablets from the preceding month, which were counted. If participants failed to return a bottle, they were asked to return it at their subsequent monthly visit and were instructed not to take any more tablets from it. At each visit, participants were also asked about self-reported adherence.

### Adherence Calculations

We used pill count data from the Mwanza and HPTN 039 trials to analyze adherence ‘coverage’, defined as tablet-taking during the entire time the participant was in the trial, including missed visits and temporary treatment interruptions.

For each participant, we calculated adherence at each visit as [tablets issued at last visit − tablets returned]/[days elapsed since last visit × 2 (given the twice-daily use of the study tablets)]. For HPTN 039, bottles that were returned late were matched to the visit when they were dispensed. In the Mwanza trial, blister strips could not be matched to the visit when dispensed since that information was not recorded; thus, all returned tablets were assumed to have been dispensed at the previous visit. Adherence was set to 0 during periods of treatment interruption. We defined ‘optimal’ adherence as taking ≥90 % of expected doses in the interval between visits.

For the Mwanza trial, since the primary analysis was modified intent-to-treat that censored women at pregnancy, for the adherence calculations, we excluded visits after women were permanently withdrawn from tablets for pregnancy. In addition, for comparability with Mwanza, we censored women at pregnancy for the adherence calculations in HPTN 039; although the primary intent-to-treat analysis included periods off treatment for pregnancy. Thus, our estimates of adherence coverage exclude visits after pregnancy.

### Summary Measures to Describe Adherence During the Trial

To describe average adherence coverage during the trial, we used two summary measures commonly reported in clinical trials to describe central tendency: overall study adherence and median adherence. Overall study adherence was calculated as [total tablets taken by all participants]/[total days in study for all participants × 2]. Median adherence was calculated in two ways: (1) calculating adherence at each visit for each participant, then computing the median of per-visit adherence over all visits for all participants; and (2) calculating overall adherence for each participant, then computing the median of per-participant adherence.

To describe optimal adherence, we used three measures commonly reported in clinical trials: (1) the proportion of visits with ≥90 % adherence; (2) the proportion of person–years with ≥90 % adherence; and (3) the proportion of participants with ≥90 % adherence during their entire study participation, defined as [total tablets taken]/[total days in study × 2] ≥90 %.

### Assessment of Missing Data and Over-Adherence

In trials that use pill counts to assess adherence, participants are asked to return the pill containers (e.g. bottles, blister packs) at each visit so that any remaining tablets can be counted. The underlying assumption is that the remaining tablets reflect the number that were dispensed at the previous visit minus the number that were ingested by the participant. However, generally there will be some visits when the containers are not returned, or when fewer than the expected number of tablets are remaining, and thus adherence is calculated to be >100 % (‘apparent over-adherence’). To calculate a summary measure of adherence, assumptions must be made about the number of tablets taken for those visits. We used two methods to explore the impact of these assumptions on summary measures, drawing on methods used by each trial in their primary publications.

Method 1 was based on the approach used in HPTN 039 [22], allowing adherence up to 105 % for each interval, due to imprecision in being able to ascertain timing of last dose with twice-daily dosing. If pill count data indicated adherence was 100–105 %, we assumed the participant had taken the tablets, and re-set adherence to 100 % for all calculations. However, if pill count data indicated adherence >105 %, or if tablets were not returned, we classified adherence as ‘unknown’. Intervals with unknown adherence were removed from the numerator and denominator and excluded from the calculation of summary measures; this approach assumes that the distribution of adherence in intervals when it is unknown is similar to that when it is known.

Method 2 was based on the approach used in the Mwanza trial [21], also allowing up to 5 % over-adherence (re-set to 100 % in calculations) but participants were assumed to have low adherence (70 %) in intervals when adherence was unknown (tablets not returned or adherence >105 %). This approach assumes that intervals with unknown adherence are likely to reflect periods of poor adherence. As a sensitivity analysis, we explored the impact of using a range of adherence levels, from 10 to 100 % (i.e. assuming that all pills were taken), during periods when adherence was unknown.

### Use of Biological Marker to Assess Periods with Unknown Adherence

Within each adherence category, we examined the effect of treatment arm on a biological outcome: for HPTN 039, genital ulcer disease (GUD) with HSV-2 aetiology confirmed by HSV DNA PCR, based on quarterly genital exams [22], and for Mwanza, genital HSV-2 DNA shedding at the 6, 12 and 24 month visits, when samples were collected [21]. Random-effects logistic regression was used to estimate odds ratios (OR) for the association of treatment with the detection of GUD or HSV-2 shedding at each visit. Models contained fixed effects for treatment arm, calculated adherence in the preceding interval, and their interaction, and random effects for subject.

## Results

We analysed 8,149 post-enrolment visits from 1,242 participants in the Mwanza trial and 47,243 visits from 3,140 participants in HPTN 039 (Table 1). In Mwanza, 98 % of blister packs were returned during the trial, and 95 % were returned at the next 3-monthly visit. In HPTN 039, 96 % of bottles were returned during the trial, 89 % at the next monthly visit.

In Mwanza, 247 (20 %) participants interrupted study drug, including 233 who became pregnant and were permanently discontinued from study medication. In HPTN 039, 231 participants interrupted study drug, including 183 who became pregnant (14 % of all females). Time off treatment for non-pregnancy related interruptions was 0.7 person–years in Mwanza and 13 person–years in HPTN 039.

**Fig. 1:**
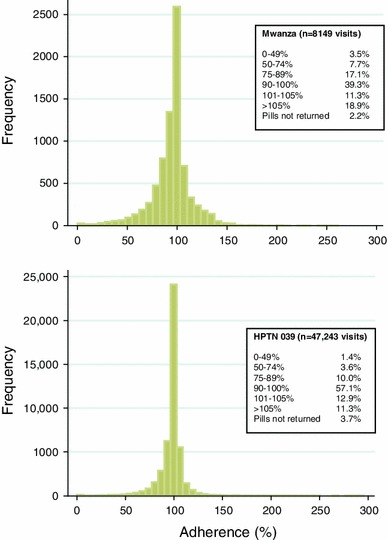
Estimated adherence at each visit in Mwanza (*top*) and HPTN 039 (*bottom*) trials among all participants (including treatment interruptions. In Mwanza, women were permanently withdrawn from study medication if pregnant, but continued with followup; the primary analysis was modified intent-to-treat that censored women at pregnancy. Therefore, for the adherence calculations, visits after women were withdrawn for pregnancy are excluded. In HPTN 039, women were temporarily withdrawn until pregnancy tests were negative, and then tablets were resumed; the primary analysis included periods off treatment for pregnancy. However, for comparability with Mwanza, visits after women were withdrawn for pregnancy are excluded from the adherence calculations and missed visits)

Figure 1 shows the distribution of adherence at each visit based on the actual pill count data. Two-thirds of participants had ≥1 interval when calculated adherence was >105 %, which accounted for 19 and 11 % of visits in Mwanza and HPTN 039, respectively.

**Fig. 2:**
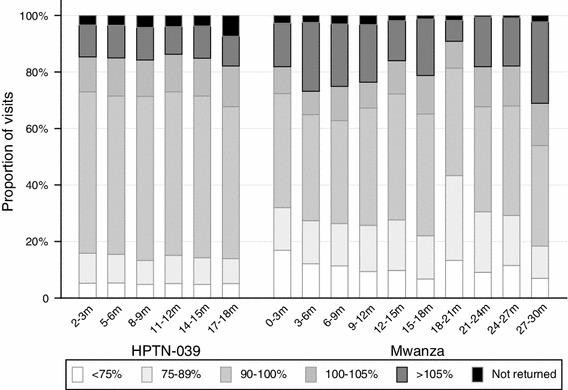
Adherence in HPTN 039 and Mwanza trials by pill counts at selected scheduled visits. Adherence in HPTN 039 shown at visits every 3 months, for the preceding month, to show data for each trial at comparable points in follow-up time

In Mwanza, where scheduled visits were quarterly, the proportion of participants with adherence 90–105 % ranged from a low of 46 % at the second visit (month 6) to a high of 57 % at month 18 (Fig. 2). In HPTN 039, with more frequent (monthly) visits, the proportion of participants with adherence 90–105 % ranged from a low of 64 % at the first visit (month 1) to a high of 73 % at month 14.

In Mwanza, participants self-reported not missing any doses in 19 % of intervals with pill count-based adherence <75 %, 6 % of intervals with adherence 75–89 %, 52 % of intervals with adherence 90–105 %, and 84 % of intervals with unknown adherence. In HPTN 039, participants self-reported not missing any doses in 38 % of intervals with adherence <75 %, 43 % of intervals with adherence 75–89 %, 83 % of intervals with adherence 90–105 %, and 80 % of intervals with unknown adherence.

### Summary Measures of Adherence

Estimates of average adherence coverage during the trials ranged from 82–98 % in Mwanza, and 88–100 % in HPTN 039, depending on the summary measure and the method used to calculate it (Table 2). Estimates of overall adherence were lower than those of median adherence. Adherence appeared highest when the median per-visit adherence was used; excluding unknown intervals, rather than considering them as periods of poor adherence, made adherence appear higher for all measures. Calculating the median as a per-participant measure gave an impression of lower adherence than calculating median adherence as a per-visit measure. Since median per-visit adherence is based on data at all visits, participants who attend more visits will contribute more information. In contrast, for median per-participant adherence, the data are first aggregated by participant, so all participants contribute equally, regardless of the number of visits attended.

**Table 2:** Commonly reported summary measures of adherence, with different assumptions regarding intervals with unknown adherence owing to missing tablet counts and apparent over-adherence (>100 %)

	Mwanza trial						HPTN 039					
	Unknown excluded^a^	Unknown replaced as 70 %^b^	Unknown replaced as^c^				Unknown excluded^a^	Unknown replaced as 70 %^b^	Unknown replaced as^c^			
			10 %	30 %	50 %	100 %			10 %	30 %	50 %	100 %
Measures of ‘average’ adherence coverage												
Median per-visit adherence^d^	95 %	90 %	90 %	90 %	90 %	98 %	99 %	97 %	97 %	97 %	97 %	100 %
Median per-participant adherence^e^	89 %	83 %	70 %	74 %	79 %	91 %	95 %	91 %	84 %	86 %	89 %	96 %
Overall adherence^f^	86 %	82 %	71 %	75 %	79 %	88 %	92 %	88 %	80 %	82 %	85 %	91 %
Measures of ‘optimal’ adherence coverage												
Proportion of visits with adherence ≥90 %	64 %	51 %	51 %	51 %	51 %	71 %	82 %	70 %	70 %	70 %	70 %	84 %
Proportion of person years with adherence ≥90 %	61 %	48 %	48 %	48 %	48 %	69 %	79 %	67 %	67 %	67 %	67 %	82 %
Proportion of participants with ≥90 % adherence^g^	48 %	27 %	18 %	19 %	21 %	56 %	73 %	54 %	34 %	39 %	45 %	74 %

^a^Unknown excluded: allow up to 105 % adherence (re-set to 100 %), otherwise unknown; exclude intervals with unknown adherence from numerator and denominator (Method 1)

^b^Unknown replaced as 70 %: allow up to 105 % adherence (re-set to 100 %), otherwise unknown; assume adherence in unknown intervals is low (70 %) (Method 2)

^c^Sensitivity analysis using a range of adherence levels to replace intervals when adherence is unknown

^d^Adherence calculated at each visit for each participant as (tablets taken since last visit/days elapsed since last visit × 2), then median taken over all visits and participants

^e^Adherence calculated overall for each participant as (total tables taken/total days in study × 2), then median taken over all participants

^f^Total tablets taken by all participants/total days in study for all participants × 2

^g^Adherence for each participant calculated as (total tablets taken during study/total days in study × 2)

Estimates of optimal adherence (≥90 %) ranged from 27–71 % in Mwanza and 56–84 % in HPTN 039, depending on the method used and whether data were first aggregated by participant before the measure was calculated. Optimal adherence appeared highest when the proportion of visits with adherence ≥90 % was used, and unknown intervals were excluded. In contrast, reporting the proportion of participants with adherence ≥90 %, with unknown intervals assumed to represent poor adherence, gave the most pessimistic picture of optimal adherence.

In both trials, participants who attended more visits had higher adherence. In Mwanza, participants’ overall adherence during the trial was on average 2.4 % higher for each one-visit increase in total visits attended. In HPTN 039, participants’ overall adherence was 1.6 % higher for each one-visit increase in total visits attended. Therefore, per-visit measures gave a better reflection of average and optimal adherence than per-participant measures.

### Impact of Assumptions Made About Missing Data and Over-Adherence on Summary Measures

Assumptions made about how to handle intervals with unknown adherence (excluding or replacing as low adherence) had a larger impact on measures of optimal adherence than on measures of average adherence. Since optimal adherence is based on the proportion of data above a fixed cut-off (≥90 %), decisions about the unknown intervals affect the denominator but not the numerator. For example, when unknown adherence was assumed to be low (≤70 %), only 48 % of person–years in Mwanza had adherence ≥90 %, compared with 61 % if unknown intervals were excluded from the calculation. In contrast, median per-visit adherence in Mwanza was similar if we assumed that adherence was low in unknown intervals or if we excluded these intervals (90 vs 95 % respectively).

The relative difference between optimal adherence obtained by the two methods of handling unknown intervals increased with the amount of missing data. Mwanza had a higher proportion of visits with unknown adherence than HPTN 039 (21 vs 15 %); therefore, the choice of method for handling unknown intervals had a larger impact on calculations of optimal adherence in Mwanza.

When we assumed adherence was low (≤70 %) in unknown intervals, most measures of optimal adherence, and median per-visit adherence, were not changed by the value assigned to the unknown intervals. In contrast, overall adherence, and median per-participant adherence, were sensitive to the value assigned, and the relative difference between the two methods of handling unknown intervals became larger as lower values were used. The proportion of participants with adherence ≥90 % was most sensitive to the value assigned to unknown intervals, with only 45 % of participants in HPTN 039 having optimal adherence if we assumed adherence was 50 % in unknown intervals, versus 54 % if we assumed adherence was 70 % in unknown intervals. At the other extreme, if we assumed adherence to be 100 % in unknown intervals, then 74 % of participants had optimal adherence. Thus, the proportion of visits with missing data and assumptions about adherence in those intervals can have a large influence on adherence estimates.

### Relationship Between Calculated Adherence and Biologic Measures of Study Product

**Fig. 3:**
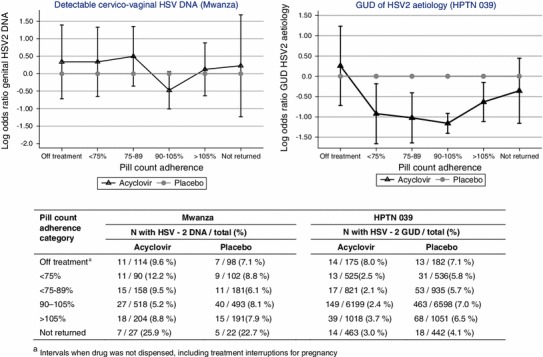
Log OR and 95 % confidence interval of genital HSV-2 DNA shedding (Mwanza, *left*) or in genital ulcer disease (GUD) of HSV-2 aetiology (HPTN 039, *right*), comparing acyclovir with placebo, within each pill count adherence category

In Mwanza, genital HSV-2 DNA shedding was detected at 176 (8 %) of 2,198 visits. In HPTN 039, HSV-2 PCR positive GUD was detected at 891 (5 %) of 18,945 visits. The association of acyclovir with reduction in genital HSV-2 shedding (Mwanza) and incident GUD (HPTN 039) was greater among participants with calculated adherence 90–105 % in the preceding interval, than among participants with calculated adherence <90 % or >105 %, or participants in whom adherence could not be calculated because tablets were not returned (Fig. 3).

## Discussion

Interpretation of efficacy in trials of user-dependent HIV prevention strategies requires clear and consistent methods for measuring and reporting adherence to study product. Our results show that calculated adherence based on pill counts varies substantially, depending on the assumptions made about how to handle missing and inaccurate data and the choice of summary measure, and can provide substantially different impressions of product use. Recent HIV prevention trials have reported adherence to study product of 90 % or higher based on pill counts [3, 4, 7]. However, such estimates may provide a simplistic or overly high indicator of adherence. Our analysis depicts a considerably more nuanced picture of adherence and emphasizes the need for more standardised approaches to reporting adherence data in order to facilitate interpretation of trial results and comparisons across trials of similar interventions.

Both HPTN 039 and Mwanza reported median adherence >90 % in their main trial publications [21, 22], but used different assumptions in their calculations so the measures were not comparable. We found that, when calculated in the same way with the same defined exclusion periods, median and overall adherence were very similar in both trials. However, measures of optimal adherence were substantially higher in HPTN 039, suggesting much higher levels of actual product use. The lack of an overall effect on biological markers of genital herpes reactivation, as reported in Mwanza [21], despite very high median adherence, shows that median adherence may not provide a very useful indicator of total study drug exposure during a trial. Summaries of optimal adherence, such as the proportion of person–years with adherence ≥90 %, may give a better reflection of actual product use, but are more sensitive to assumptions made about intervals with unknown adherence, particularly when the amount of missing data is large. Another disadvantage of measures of optimal adherence is that they dichotomise participants based on a fixed threshold value believed to represent sufficient drug exposure to achieve a therapeutic result; however, in practice, there are a range of factors that determine the threshold that is needed for a satisfactory treatment effect. Furthermore, as a review of adherence measurement points out, adherence in the 90–100 % range is generally considered ‘good’, but this figure is not inconsistent with multi-day lapses in dosing during which drug concentrations fall below therapeutic levels, particularly with drugs with a short half-life like acyclovir [25].

Adherence in a particular interval can be unknown for several reasons: the participant may fail to return the tablets (missing data), calculated adherence may be >100 %, or the tablets may be returned late, so a participant is issued with a new set of tablets before returning the first set. In Mwanza, we could not link tablets to the visit dispensed, so we assumed that all tablets returned were issued at the previous visit. In HPTN 039, a unique bottle identification number was recorded when tablets were dispensed and returned, so we were able to match the bottles to the visit dispensed. Thus, unique study product identifiers may offer important advantages for assessing the ‘measurability’ of adherence in a trial. In addition, shorter times between visits may help reduce errors by reducing the amount of surplus tablets issued and allowing adherence messages to be reinforced more frequently. Mwanza had longer intervals between visits and was of longer duration than HPTN 039, which may have resulted in more intervals with unknown adherence. The proportion of visits with unknown adherence is a key factor influencing adherence estimates, and should be a greater focus in implementation of future trials and critiqued carefully when trials report their results.

In both trials, although the biological markers of genital herpes reactivation were different (PCR confirmed GUD in HPTN 039 and HSV-2 shedding ascertained at 3 time points in Mwanza), we found that the effect of acyclovir on biologic measures of anti-HSV-2 activity was similar among participants with pill counts that suggested adherence >105 % and those with adherence <90 %, and lower than those with 90–105 % adherence. This is consistent with recently-reported findings from the Partners in Prevention HSV/HIV Transmission Study, in which low and apparent over-adherence to acyclovir in HIV/HSV-2 dually infected persons was associated with a reduced effect on HIV-1 plasma viral load and HSV-2 GUD [26]. Acyclovir suppression significantly but incompletely reduces symptomatic genital ulcers and HSV-2 shedding, so both are imperfect markers of adherence and drug exposure [27]. However, since clinical effects such as genital ulcers are determined by adherence over a period of time, the incidence of GUD in HPTN 039 and HSV-2 shedding in Mwanza may provide a clinical measure of acyclovir exposure in these trials. Our findings suggest that participants with tablet counts indicating over-adherence are not actually taking the tablets; similar findings have been reported from studies of hypertension [28] and anti-obesity medication [29], and a study that used phenobarbital as a chemical marker of adherence [30]. Thus, when calculating summary measures, it may be better to assume that intervals with apparent over-adherence represent periods of low adherence, rather than excluding them or considering them as if they reflect 100 % adherence. Reporting the proportion of visits with apparent over-adherence may provide important information about actual product use.

Although tablet counts suggesting over-adherence beyond a certain cut-off (e.g., 105 %) could represent pill dumping by participants to improve their apparent adherence, some shortfall in the number of tablets returned should be allowed, to account for the occasional dropped tablet or imprecision in accounting for tablets that were taken on the day dispensed and returned. There could also be some genuine over-adherence, for example, if the participant forgets she or he has taken a tablet and takes an extra one. Using a fixed number of tablets to define over-adherence, rather than a percentage, could be a more reasonable approach, particularly with longer intervals between visits.

In addition to potential bias from intervals with unknown adherence, the choice of summary measure used to report trial adherence can provide a misleading estimate of product use. As our results demonstrate, reporting the median, either per-visit or per-participant, to summarise trial adherence can obscure a large number of subjects with poor adherence. In both trials, participants who attended more visits were in the trial for longer and had better adherence than those who attended less frequently. Thus summary measures that gave equal weight to the participants who dropped out early and those who remained in the trial provided an impression of lower adherence than measures that were weighted by the number of visits attended. This further highlights the need for a standardisation in calculating and reporting summary measures.

In both trials, we found that adherence based on self-report of number of missed doses was higher than that based on pill counts. We did not use self-report in our adherence calculations for this paper; however, self-report was used to supplement the pill counts in the adherence calculations for the main trial papers. The tendency of self-report to overestimate adherence, when compared with pill counts [3, 6], electronic monitoring devices [31] or drug detection [2, 6, 32] methods has been cited by other studies. Although asking participants about the number of missed pills may be of limited utility, other methods of self-evaluation, such as qualitative rating scales, may provide more reliable information [33].

Strengths of our study include analyses of data from two large HIV prevention efficacy trials of acyclovir suppression, with the same intervention but different visit schedules and different procedures for performing tablet counts. This allowed us to examine the impact of different study procedures and analysis assumptions on reported measures of adherence. Limitations of our study include that pill counts of returned study product are an imperfect method of assessing adherence, and did not allow us to assess finer patterns of adherence, for example, as would be possible with electronic daily monitoring. A further limitation is that clinic-based pill counts have been found to overestimate adherence compared with electronic monitoring [34, 35] or drug levels [6], and are subject to manipulation, as participants may discard unused pills to create an illusion of good adherence. The validity of pill counts to assess adherence relies on the assumption that unreturned tablets were ingested by the participant; however, estimates of adherence may be severely biased if unreturned tablets do not reflect actual product use. An additional limitation is that pill counts were done at every visit; however, GUD and HSV-2 shedding were measured less frequently. Although we had some clinical indicators of adherence, the half-life of acyclovir is ~3 h with minimal accumulation [36], thus limiting acyclovir drug levels as a gold standard for evaluating our adherence measures using pill counts. Lastly, the Mwanza trial and the majority of the HPTN 039 sites were in resource-limited settings; therefore, our finding that pill counts suggestive of over-adherence are likely to represent pill dumping may not be generalisable to other settings.

Our results have demonstrated that there is a clear need for standardisation in adherence reporting. The CONSORT statement was developed to provide a set of guidelines for the clear, complete and transparent reporting of results from randomised controlled trials [37–39]. Although these guidelines implicitly recognise the importance of adherence in interpreting trial results, there are no recommendations for how to report adherence. For clinic-based pill counts of unused study product, authors should report the proportion of visits in which tablets were not returned, or where pill counts found adherence to be >100 %, and how intervals with unknown adherence were handled in calculation of summary measures. To provide a measure of uncertainty of these adherence metrics, it is useful to report the summary measures using different assumptions (e.g. excluding unknown intervals and considering these as low adherence). We recommend that trials should report measures of study medication adherence coverage (i.e. including missed visits and time off treatment), as the most relevant measure for understanding results from the intent-to-treat analysis. Although all summary measures provide an imperfect assessment of adherence, overall adherence, since it takes into account the total days that each participant is in the trial, may provide a reasonable reflection of drug exposure on average over the duration of the trial. Some estimate of the amount of person–time with optimal adherence may be useful, since this relates exposure to levels needed to achieve a biological effect. However, in addition to the problems of dichotomising adherence as discussed above, there can be difficulties in operationalising ‘optimal’ adherence: the definition is likely to differ between study drugs, formulations and dosing regimens (and may not be known for new investigational products), creating further complications for standardising definitions across trials. Summary measures of median adherence, either per-visit or per-participant, are not particularly informative.

In summary, we have shown that summary measures of adherence in two recent HIV prevention trials of acyclovir varied considerably depending on assumptions and analysis method. Our findings of a relationship between adherence and clinical markers (GUD or HSV-2 genital shedding) indicate that intervals of apparent over-adherence are likely to represent pill-dumping. Thus, the proportion of visits with unknown adherence, either because tablets are not returned or calculated adherence is too high, may provide important information. The impact of the assumptions made on calculated measures indicate the important need for standardisation of best practices in adherence reporting, to aid in the interpretation and understanding of user-dependent HIV prevention interventions.
